# Influence of lower-limb mechanical axis on the curative effect of medial high tibial osteotomy for knee osteoarthritis

**DOI:** 10.1186/s12893-022-01629-5

**Published:** 2022-05-13

**Authors:** Long Yuan, Shuaishuai Niu, Chuanxing Zhai, Sen Li, Jichao Bian, Xiaowei Zhao, Yuanmin Zhang, Guodong Wang

**Affiliations:** 1 Department of Clinical, Jining Medical University, Jining, Shandong China; 2 Department of Orthopedics and Joints, Affiliated Hospital of Jining Medical University, Jining, Shandong China

**Keywords:** High tibial osteotomy, Tinetti assessment score, Lower-limb mechanical axis

## Abstract

**Purpose:**

To investigate the effect of the ratio of the medial tibial plateau width to the total tibial plateau width on the therapeutic efficacy of high tibial osteotomy (HTO) on the medial side for the treatment of knee osteoarthritis.

**Methods:**

In this study, we retrospectively analyzed information of 278 patients who underwent medial HTO for knee osteoarthritis with varus deformity. The Tinetti Gait and Balance Assessment Tool, the Visual Analog Scale (VAS), and the Knee Society Scoring System (KSS) were used to comprehensively evaluate the function of the knee joint after HTO.

**Results:**

After adjusting for potential confounding factors (i.e., age, gender, body mass index/BMI, and surgical site), the Tinetti assessment score was optimized when the degree of correction was 53.67%, with the β-value on the left and right sides of the inflection point of 0.49 (confidence interval, CI: 0.20, 0.78, P = 0.0009) and− 0.26 (95% CI: − 0.30, − 0.22, P < 0.0001), respectively. The KSS score was optimized when the degree of correction was 55.45%, with the β-value on the left and right sides of the inflection point of 2.77 (95% CI: 1.64, 3.90, P < 0.0001) and − 1.18 (95% CI: − 1.46, − 0.91, P < 0.0001), respectively. The VAS score was the lowest when the degree of correction was 55.00%, with the β-value on the left and right sides of the inflection point of − 0.16 (95% CI: − 0.29, − 0.03, P = 0.0146) and 0.08 (95% CI: 0.05, 0.10, P < 0.0001), respectively. Stratified analysis showed that the BMI affected the Tinetti assessment score (β =  − 0.14, 95% CI: − 0.24, − 0.04, P = 0.0071). According to the smooth-curve fitting results, when the BMI was > 28, the Tinetti assessment score showed a negative trend.

**Conclusion:**

The degree of lower-limb mechanical axis correction correlated with the functional status of the knee joint after MOW HTO. When the ratio of the medial tibial plateau width to the total tibial plateau width was approximately 55%, the post-MOW HTO outcomes were optimized and the patients experienced the highest satisfaction. In addition, very high BMI was not conducive for the postoperative recovery of the knee joint function.

*Level of evidence:* III Case–control study/Retrospective comparative study.

## Introduction

Osteoarthritis is one of the most common joint diseases in adults, with the main pathological features of gradual degradation of articular cartilage, osteophyte formation, and synovial hyperplasia, leading to joint pain, loss of joint function, and even disability. Because the direction of external force and limb orientation produce knee adduction moments during walking in humans, knee osteoarthritis with varus deformity is the most prevalent form of osteoarthritis [1]. The knee adduction torque is closely related to the occurrence and progression of knee osteoarthritis, which is the theoretical basis for the strategy of treating knee osteoarthritis with varus deformity by correcting the lower-limb mechanical axis under the premise of preserving the knee joint [2].

Since the high tibial osteotomy (HTO) was first reported by Jackson et al. in 1961, many orthopedic surgeons have started to perform this surgery [3]. It is a preferred procedure for patients with knee osteoarthritis with varus deformity at a relatively young age and with higher exercise needs [4]. The specific implementation of HTO includes a variety of surgical methods, among which a relatively common method is medial opening wedge high tibial osteotomy (MOW HTO) [5]. MOW HTO is a valgising procedure to correct a varus deformity of the knee. The point where the lower-limb mechanical axis intersected the tibial plateau tangent line was called the Mikulicz point[6]. MOW HTO adjusts the position of the Mikulicz point, thereby alleviating the medial gap pressure on the knee joint and delaying the progression of osteoarthritis. Many orthopedic surgeons choose MOW HTO for moving the Mikulicz point to the lateral edge of the midpoint of the tibial plateau tangent line. There is differing opinion of the ideal intended correction with suggested corrections for Mikulicz point adjusted to Fujisawa’s point (67.5%), Dugdale (62%), Feller (58%) or neutral at (50%) or other target ranges[7, 8]. At best these varying target points or ranges are based upon opinion without any reference to patient recorded outcomes. The literature revealed that the Fujisawa point is still controversial and lacks clinical evidence [9]. Amis et al. indicated that it is impossible to find sufficient mechanical principles and evidence to support the Fujisawa point, which seems to be a subjective judgment [10].

Based on the above controversy, in this study, we collected the medical reports of 278 patients with knee osteoarthritis with varus deformity. Three outcome measures were recorded: the preoperative and postoperative Tinetti Gait and Balance Assessment Tool, the Visual Analog Scale (VAS), and the Knee Society Scoring System (KSS). We explored how the corrected lower limb mechanical axis influenced these outcome measures and explored the influence of BMI on the outcome scores as a secondary aim. The correction degree in this study refers to the ratio of medial tibial plateau width to the total tibial plateau width.

## Information and methods

### General information

**Fig. 1:**
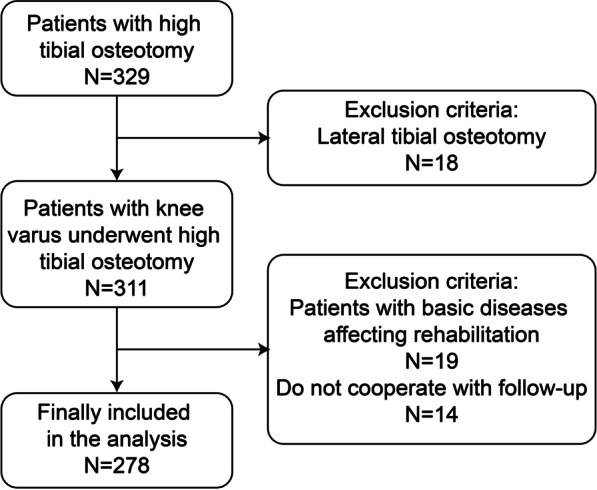
Screening protocol of the research subjects in this study

The present study retrospective observed 278 patients (113 males and 165 females) who underwent HTO for knee osteoarthritis (see Fig. 1 for the screening process) in the Department of Orthopedic Surgery, Affiliated Hospital of Jining Medical University from January 2014 to December 2018. The inclusion criteria of this study were as follows: (1) Patients with knee osteoarthritis with varus deformity of the lower limbs (varus deformity originating within the tibia); (2) patients with knee joint pain caused by osteoarthritis with VAS ≥ 6; (3) patients with restricted knee joint function due to osteoarthritis, leading to < 500 m walking distance on level ground; and (4) patients with restricted knee joint flexion and extension function, leading to inability to squat normally and climb stairs (up and down); thus, seriously affecting their daily life. The exclusion criteria of this study were as follows: (1) patients with varus deformity of the lower limbs originating within the knee joint (joint line convergence angle > 3º); (2) patients with inflammatory knee joint diseases (e.g., rheumatoid arthritis); (3) patients with excessively lose knee joint or another underlying disease (s) that would affect the postoperative joint exercise; (4) patients that fail to or poorly follow the medical instructions; and (5) patients with lateral closing wedge high tibial osteotomy(LCW HTO). The research protocol of this study was approved by the Ethics Committee of Affiliated Hospital of Jining Medical University (approval number: 2021C015).

### Surgical methods

After the patient entered the operation room, the anesthesiologists, surgeons, and itinerant nurses together evaluated the patient information and the correctness of the surgical site. Patients were positioned supine for general anesthesia combined with peripheral nerve blocks. After the successful induction of anesthesia, routine skin disinfection, surgical draping, and the application of adhesive membrane were performed to prevent postoperative infection at the surgical site. The conventional medial and lateral parapatellar portals were employed for arthroscopy to explore the morphology and tension of the synovium, articular cartilage, medial and lateral menisci, and the anterior and posterior cruciate ligaments. Any lesions identified at arthroscopy were removed or repaired. An incision of the proximal medial lower leg was performed, followed by the separation of the tissues in layers, to expose the proximal tibia. Two positioning K-wires were inserted from the upper edge of the pes anserinus to the tip of the fibular head. The locations of the positioning wires were confirmed by intraoperative fluoroscopy, followed by cutting the bone along the cross-section of the positioning wires to save approximately 1 cm of the lateral cortical hinge. A coronal osteotomy was subsequently performed behind the tibial tubercle. A lamina spreader was used to open the cross-section of the osteotomy gap successively and slowly for the determination of the lower-limb mechanical axis, followed by inserting a locking plate into the medial tibial region and securing four screws at the proximal end and another four screws at the distal end. Based on the practical expansion angle, the autogenous iliac crest bone graft was placed, and fluoroscopy was performed again with alignment rod to evaluate the internal fixation position and the lower-limb mechanical axis. After washing with normal saline, the incisions were sutured in layers. The operation conducted in the present study was performed by only one surgeon (Dr. Wang).

### Observation indexes

The primary index observed in this study was the Tinetti Gait and Balance Assessment score, with the scoring content including sitting balance, rises from the chair, attempts to rise, standing balance (first 5 s), standing balance, nudged (the assessment subject should stand with feet as close together as possible, after which the examiner pushes the subject lightly three times), eyes closed (same as in the previous step), turning 360 degrees, sitting down, an indication of a gait, step length and height, step symmetry, step continuity, path (walking distance is approximately 3 m), trunk stability, and walking stance. The maximum score of the Tinetti Gait and Balance Assessment is 28 points; the higher the total score, the better is the gait and balance function of the knee joint.

The secondary indices were (1) VAS—VAS assessment of a patient’s pain by scoring, with the maximum score of 10 points, where the score 0 represents painless and the score 10 represents unbearable and severe pain, while (2) KSS—KSS assesses the postoperative recovery of the knee joint function in the following aspects: knee pain, range of motion, stability, flexion deformity, hyperextension, the position of the lower-limb mechanical axis, and resting pain. The maximum score of KSS is 100 points; the higher the total score, the better is the overall function of the knee joint.

### Statistical analysis

Continuous variables were expressed as mean ± standard deviation (normal distribution) or median (quartile) for a skewed distribution, and categorical variables were expressed as frequency or percentages. One-way analysis of variance (ANOVA) (normal distribution), the Kruskal–Wallis H test (skewed distribution), and the Chi-square test (categorical variables) were employed to compare the means and proportions between three groups which were selected by the analysis software as an equal division of continuous variables. A univariate linear regression model was applied to evaluate the associations between the correction degree and the Tinetti assessment score/VAS/KSS. We also used the generalized additive model (GAM) to determine non-linear relationships. If a non-linear correlation was recorded, a piecewise linear regression model was used to calculate the threshold effect of the correction degree on the Tinetti assessment score/VAS/KSS. When the ratio between the correction degree and the Tinetti assessment score/VAS/KSS appeared to be in a smoothed curve, the recursive method automatically calculated the inflection point. Subgroup analyses were performed using stratified linear regression models. The modifications and interactions of the subgroups were inspected by the likelihood-ratio test. All analyses were performed using the R language (http://www.R-project.org, The R Foundation) and the statistical software EmpowerStats (http://www.empowerstats.com, X&Y Solutions, Inc., Boston, MA). P < 0.05 (two-sided) was considered to indicate statistical significance.

## Results

### General results

Among the 278 patients included in this study, the mean age was 56.51 ± 8.60 years; the mean BMI was 27.01 ± 2.67 kg/m^2^, and 60.9% of the patients were females. Table 1 presents the baseline characteristics of the patients. No significant differences in age, body mass index (BMI), gender, surgical site (left/right knee), or medial proximal tibial angle (MPTA) were found between the groups of different angles (This is EmpowerStats' automatic equal division of continuous variables into 3 groups based on its software internal protocol).

**Table 1:** Baseline characteristics of participants (N = 278)

Characteristic	The correction degree tertiles (%)			*P*-value
	T1 (≥ 50.01– < 54.98)	T2 (≥ 54.98– < 59.56)	T3 (≥ 59.56– < 86.02)	
No. of participants	87	96	95	
Age (years, mean ± sd)	58.02 ± 9.01	55.41 ± 8.54	56.24 ± 8.15	0.112
BMI(kg/m^2^, mean ± sd)	27.01 ± 2.67	27.37 ± 3.35	27.32 ± 3.48	0.724
Gender (n, %)				0.682
Female	53 (60.9%)	59 (61.5%)	53 (55.8%)	
Male	34 (39.1%)	37 (38.5%)	42 (44.2%)	
Leg (n, %)				0.882
Right	44 (50.6%)	45 (46.9%)	46 (48.4%)	
Left	43 (49.4%)	51 (53.1%)	49 (51.6%)	
MPTA(°)	82.36 ± 3.12	83.57 ± 2.05	82.55 ± 3.38	0.673

*BMI* body mass index, *MPTA* medical proximal tibial angle

### Univariate analysis

As presented in Table 2, the results of the univariate analysis indicated significant differences in BMI and degree of correction among the patients with different Tinetti assessment scores. BMI was not significantly different between patients with Tinetti assessment scores Middle and Low (P = 0.5506). The BMI of patients with score High was lower than the BMI of patients with score Low by 1.10 (95% CI: − 1.90, − 0.30, P = 0.0071), which suggests a non-linear relationship between BMI and Tinetti assessment score and no correlation between the BMI, VAS and KSS scores. The degree of correction of patients with score Middle was higher than that of the patients with score Low by 1.01 (95% CI: 0.42, 1.60, P = 0.0009). The degree of correction of patients with score High was lower than that of the patients with score Low by 3.44 (95% CI: − 4.03, − 2.85, P < 0.0001), which indicates that the degree of correction and the Tinetti assessment score had a segmented effect. The KSS score was similar to the Tinetti assessment score with respect to its relationship with the degree correction. However, the VAS score of patients with Tinetti assessment score Middle was lower than that of the patients with Tinetti assessment score Low by 0.87 (95% CI: − 1.24, − 0.50, P < 0.0001). The VAS score of patients with Tinetti assessment score High was higher than that of the patients with Tinetti assessment score Low by 1.02 (95% CI: 0.64, 1.39, P < 0.0001). No significant differences in age, gender, or surgical site (left/right knee) were observed among patients with different Tinetti assessment, VAS, or KSS scores.

**Table 2:** Univariate analysis for Tinetti assessment score/VAS/KSS

Covariate	Statistics	Tinetti Assessment Score		VAS		KSS	
		β (95%CI)	*P-*value	β (95%CI)	*P-*value	β (95%CI)	*P-*value
Age (years, mean ± sd)	56.51 ± 8.60	0.01 (− 0.03, 0.04)	0.7846	0.02 (− 0.00, 0.04)	0.0599	− 0.01 (− 0.22, 0.21)	0.9543
Low (n, %)	93 (33.45%)	Ref.		Ref.		Ref.	
Middle (n, %)	88 (31.65%)	0.29 (− 0.53, 1.11)	0.4887	0.24 (− 0.19, 0.67)	0.2790	3.83 (− 0.74, 8.39)	0.1014
High (n, %)	97 (34.89%)	0.40 (− 0.40, 1.20)	0.3282	0.49 (0.06, 0.91)	0.0251	− 0.20 (− 4.65, 4.25)	0.9299
BMI(kg/m2, mean ± sd)	27.24 ± 3.19	− 0.14 (− 0.24, − 0.04)	0.0071	0.03 (− 0.02, 0.09)	0.2591	0.28 (− 0.86, 0.30)	0.3384
Low (n, %)	90 (32.37%)	Ref.		Ref.		Ref.	
Middle (n, %)	92 (33.09%)	− 0.25 (− 1.05, 0.56)	0.5506	0.15 (− 0.28, 0.59)	0.4888	− 4.26 (− 8.80, 0.29)	0.0676
High (n,%)	96 (34.53%)	− 1.10 (− 1.90, − 0.30)	0.0071	0.41 (− 0.02, 0.84)	0.0618	− 3.80 (− 8.30, 0.70)	0.0993
Gender (n, %)							
Female	165 (59.35%)	Ref.		Ref.		Ref.	
Male	113 (40.65%)	− 0.27 (− 0.94, 0.40)	0.4232	0.06 (− 0.30, 0.42)	0.7613	2.79 (− 0.97, 6.54)	0.1467
Leg (n, %)							
Right	135 (48.56%)	Ref.		Ref.		Ref.	
Left	143 (51.44%)	0.29 (− 0.36, 0.95)	0.3808	− 0.19 (− 0.54, 0.16)	0.2946	0.17 (− 3.53, 3.87)	0.9297
Corrrection degree (%, mean ± sd)	59.02 ± 7.40	− 0.22 (− 0.26, − 0.18)	< 0.0001	0.05 (0.03, 0.08)	< 0.0001	− 0.68 (− 0.91, − 0.44)	< 0.0001
50.01–54.97%	87	Ref.		Ref.		Ref.	
54.98–59.55%	96	1.01 (0.42, 1.60)	0.0009	− 0.87 (− 1.24, − 0.50)	< 0.0001	15.83 (12.49, 19.18)	< 0.0001
59.56–86.02%	95	− 3.44 (− 4.03, − 2.85)	< 0.0001	1.02 (0.64, 1.39)	< 0.0001	− 9.69 (− 13.04, − 6.33)	< 0.0001

### Stratified analysis

**Fig. 2:**
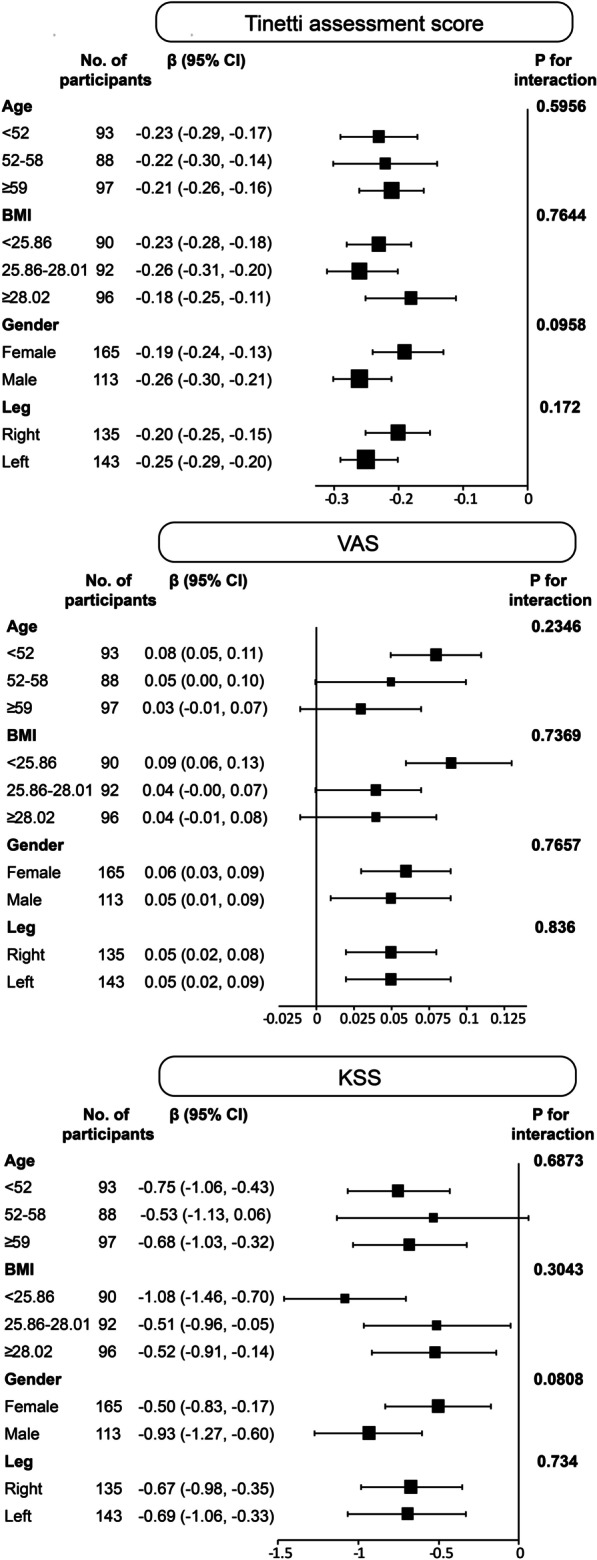
Stratified analysis forest plot

As shown in Fig. 2, stratified analyses of age, BMI, gender, and surgical site (left/right knee) revealed that the Tinetti assessment score was stable among the abovementioned four variables (P values for interaction were 0.5956, 0.7644, 0.0958, and 0.1720, respectively). The results of the accompanying assessment indexes, VAS score, and KSS score, were equally stable across each layer (P values for interactions were 0.2436, 0.7369, 0.7657, and 0.8360; 0.6873, 0.3034, 0.0808, and 0.7340, respectively).

### Smooth-curve fitting and analysis of threshold effects

**Fig. 3:**
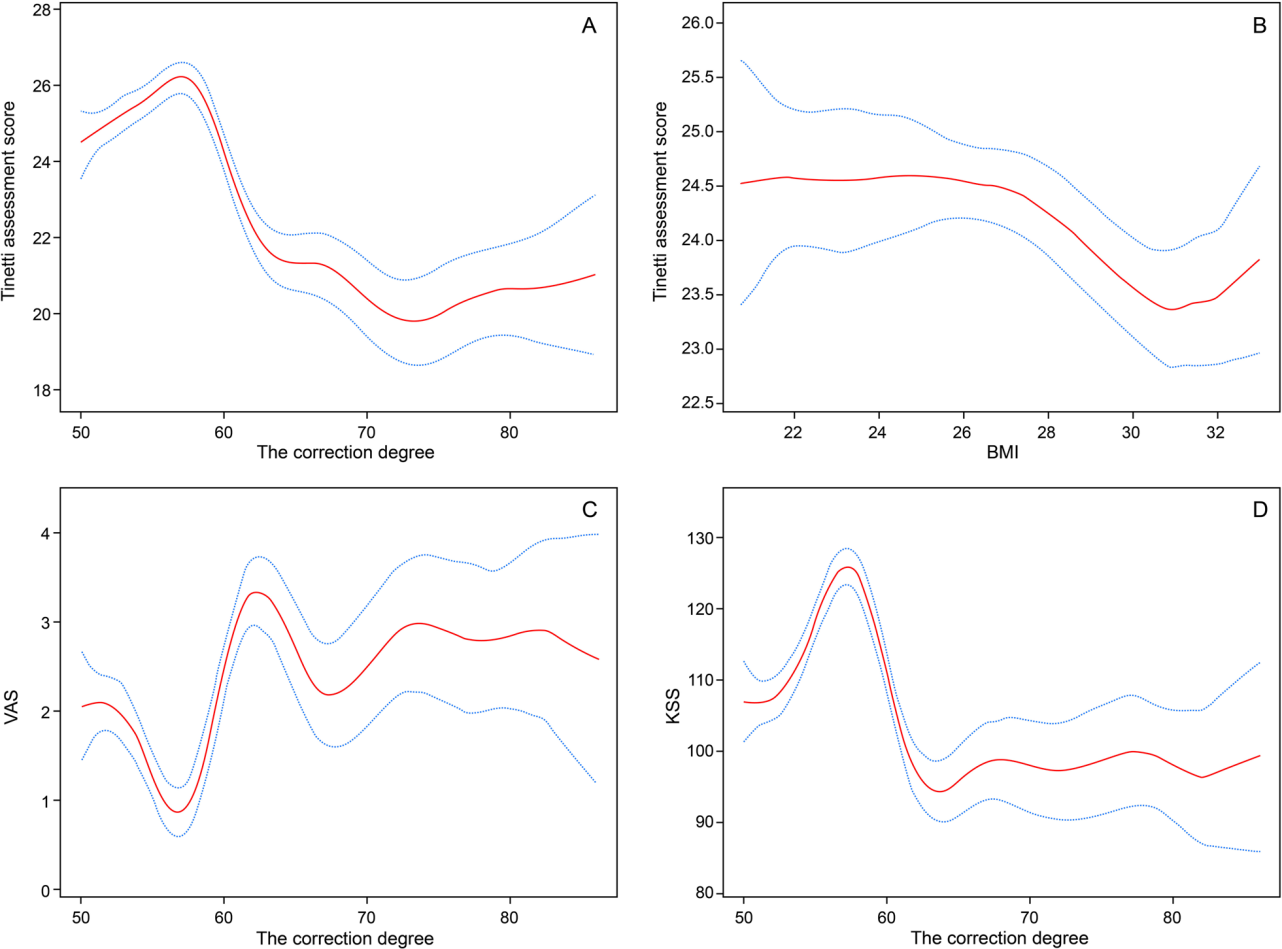
Association between the correction degree and Tinetti assessment score/VAS/KSS and association between the BMI and Tinetti assessment score. In each plot, the solid red line represents the smooth curve fit between the variables, while the blue bands represent the 95% confidence interval from the fit. **a** A threshold, nonlinear association between the correction degree and Tinetti assessment score was detected (P < 0.001) in a generalized additive model (GAM). Adjusted for age, gender; BMI, and leg. **b** A threshold, nonlinear association between BMI and Tinetti assessment score was detected (P < 0.001) in the GAM. Adjusted for age, gender, the correction degree, and leg. **c** A threshold, nonlinear association between the correction degree and VAS was detected (P < 0.001) in the GAM. Adjusted for age, gender, BMI, and leg. **d** A threshold, nonlinear association between the correction degree and KSS was detected (P < 0.001) in the GAM. Adjusted for age, gender, BMI, and leg.

Figure 3 presents the results of smooth-curve fitting, and Fig. 3 presents the fitting curve of the degree correction and the Tinetti assessment score, VAS and KSS. The trend of the curve was consistent with the results of univariate analysis and regression analysis. An analysis of threshold effect is presented (Table 3) where the Tinetti assessment score was highest at 53.67%, the KSS was highest at 55.45% and the VAS pain score was lowest at 55.00%. The results were mutually complete with the univariate analysis and the regression analysis. Figure 3b presents the smooth-curve fitting between the BMI and the Tinetti assessment score.

**Table 3:** Threshold effect analysis of the correction degree and Tinetti assessment score/VAS/KSS using piecewise linear regression

Infection point	Effect size (β)	95%CI	*P*-value
Tinetti assessment score			
< 53.67	0.49	(0.20, 0.78)	0.0009
≥ 53.67	− 0.26	(− 0.30, − 0.22)	< 0.0001
VAS			
< 55	− 0.16	(− 0.29, − 0.03)	0.0146
≥ 55	0.08	(0.05, 0.10)	< 0.0001
KSS			
< 55.45	2.77	(1.64, 3.90)	< 0.0001
≥ 55.45	− 1.18	(− 1.46, − 0.91)	< 0.0001

Effect: The correction degree. Cause: Tinetti assessment score/VAS/KSS

All adjusted for age, gender, BMI, and leg

## Discussion

The main findings of this paper were improved outcome measures when the mechanical axis was corrected to 53.67–55.45%. For simplicity we would recommend a correction with Mikulicz point at 55% of the tibial width. Knee osteoarthritis is a global health problem with no cure. The degenerative changes in the articular cartilage due to aging are the leading cause of knee osteoarthritis. Osteoarthritis severely impairs quality of life and carries a significant economic burden [5]. Various studies have reported that MOW HTO has a significant therapeutic effect in medial knee osteoarthritis. MOW HTO corrects varus deformity, improves clinical symptoms and may delay the requirement for knee arthroplasty (partial or total) [11]. A minor change in the position of the lower-limb mechanical axis can lead to a significant change in the load distribution on the knee joint. Therefore, over-correction or under-correction can lead to unfavourable outcomes and reduced survival [1, 12]. However, it is the surgeon’s responsibility to select an intended correction for each individual patient [13]. A study by Mitsuaki Kubota suggests that adjusting the lower-limb mechanical axis to pass through the Fujisawa point is the ideal degree of osteotomy. A study by Staubli suggests that only a slight correction of varus deformity is required, and the degree of correction should not exceed 5% and the Fujisawa point. A study by Sung-SahnLee reported that the lower-limb mechanical axis could pass through the lateral edge of the tibial intercondylar eminence to achieve satisfactory surgical outcomes and also simplify the process of assessing the position of the lower-limb mechanical axis [10, 14–16]. Therefore, the degree of correction is still controversial in the existing literature. This paper is the first to show evidence of improved patient outcomes at the specific correction of 55%

The performance-oriented mobility assessment has become a widely used evaluation tool for assessing balance and mobility in the elderly since its introduction in 1986 by Tinetti et al. [17]. The advantages of the performance-oriented mobility assessment are that it includes both balance and gait components and its reliability and effectiveness have been clinically recognized in various neurological diseases [18]. Parveen et al. used the performance-oriented mobility assessment in patients with knee arthritis, which lead to high interrater reliability; thus, confirming the suitability of the assessment for increasing the movement and balance ability in patients with knee arthritis [19]. In this study, the Tinetti assessment score, VAS and KSS are all outcomes scores. To better improve the generalisabilityof the research results, this study also collected the VAS and KSS of all patients to reevaluate the postoperative satisfaction and knee joint function of all patients.

In this study, the position of the lower-limb mechanical axis had a significant impact on the Tinetti assessment score, the VAS score, and the KSS score of the patients. Adjustment of the lower-limb mechanical axis to the lateral side in patients with osteoarthritis combined with varus deformity in the knee helped to shift the pressure of the knee joint to the lateral compartment; thus achieving the purpose of the treatment [20, 21]. If the degree of adjustment of the lower-limb mechanical axis to the lateral side was under-correction, the pressure load of the medial compartment would not be fully relieved, and the postoperative pain and the postoperative knee joint function of the patients would not achieve satisfactory results. However, if the degree of adjustment of the lower-limb mechanical axis to the lateral side was over-correction, the wedge-shaped osteotomy designed intraoperatively would be wider, causing the hinge points to become extremely weak; thus, greatly increasing the risk of tibial fracture during osteotomy [22]. Over-correction would abnormally increase the pressure on the lateral compartment of the knee joint. The patients can develop lateral compartment osteoarthritis [23] and obvious valgus deformity of the knee joint. Patients tend to dislike the aesthetics of a valgus deformity.

The present study also reported that the BMI of the patients affected the Tinetti assessment score. However, no significant relationship was observed between the patient’s BMI, the VAS score, and the KSS score. In patients with obesity, due to their excess weight, the pressure in the medial compartment of the knee joint is relatively high, which may limit the movement of the affected limb after surgery and not be conducive to the postoperative functional recovery of the knee joint [24]. In one study the oxford knee scores of obese patients (BMI > 30) were reduced, and in the present study the Tinsetti assessment score was reduced for patients with BMI > 28 [25].

The present study has some limitations. First, since this was a retrospective observational cross-section study, we only observed the relationship between the lower-limb mechanical axis position and the knee joint function but did not determine whether there was a causal relationship between the two. Second, this study was based upon a specific localized population and so the generalizability of the results is currently not defined, we would suggest that this study be repeated on other study populations. Future research is required to expand the sample size and population range. Finally, this study did not discuss the patient’s education level, genetic factors, and environmental factors, which need to be considered in future research.

## Conclusion

The lower-limb mechanical axis position had a non-linear correlation with the functional status of the knee joint after MOW HTO. When the ratio of the medial tibial plateau width (the distance between the medial edge of the tibial plateau and the intersection point of the tibial plateau tangent line and the lower-limb mechanical axis) to the total tibial plateau width was approximately 55% (instead of 67.5%), the postoperative outcomes of MOW HTO were optimized and the patients had the highest satisfaction. In addition, high BMI was not conducive for the postoperative recovery of the knee joint function.

## Data Availability

The datasets used and/or analyzed during the current study are available from the corresponding author on reasonable request.
